# Deep-sea infauna with calcified exoskeletons imaged in situ using a new 3D acoustic coring system (A-core-2000)

**DOI:** 10.1038/s41598-022-16356-3

**Published:** 2022-07-27

**Authors:** Katsunori Mizuno, Hidetaka Nomaki, Chong Chen, Koji Seike

**Affiliations:** 1 Department of Environment Systems, Graduate School of Frontier Sciences, The University of Tokyo, Kashiwanoha, Kashiwa, Chiba, 277-8561 Japan; 2 X-STAR, Japan Agency for Marine-Earth Science and Technology (JAMSTEC), 2-15 Natsushima-cho, Yokosuka, 237-0061 Japan; 3 Geological Survey of Japan, National Institute of Advanced Industrial Science and Technology (AIST), Central 7, 1-1-1 Higashi, Tsukuba, Ibaraki 305-8567 Japan; 4 Department of Natural Environmental Studies, Graduate School of Frontier Sciences, The University of Tokyo, 5-1-5 Kashiwanoha, Kashiwa, Chiba, 277-8564 Japan

**Keywords:** Ecology, Ocean sciences, Engineering

## Abstract

The deep ocean is Earth’s largest habitable space inhabited by diverse benthic organisms. Infauna play crucial roles in shaping sedimentary structures, relocating organic matter, porewater chemistry, and hence biogeochemical cycles. However, the visualization and quantification of infauna in situ inside deep-sea sediment has been challenging, due to their sparse distribution and that deep-sea cameras do not visualize animals living below the sediment surface. Here, we newly developed a 3D acoustic “coring” system and applied it to visualize and detect burrowing bivalves in deep-sea sediments. The in situ acoustic observation was conducted at a dense colony of vesicomyid clams in a hydrocarbon seep in Sagami Bay, Japan, focusing on a patch of juvenile clams with a completely infaunal life style. We clearly observed strong backscatters from the top and lower edges of animals in our 3D acoustic data. At least 17 reflectors were identified in the survey area (625 cm^2^), interpreted to correspond to living clams. The estimated depths of the lower edge of clams ranged between 41 and 98 mm. The acoustic system presented here is effective for detecting and monitoring infauna with calcified exoskeletons. This novel tool will help us better assess and understand the distribution of deep-sea infauna, particularly those groups with hard exoskeletons, as well as biogeochemical cycles.

## Introduction

Changes in the marine environment due to anthropogenic activities, such as ocean acidification, eutrophication, deoxygenation, and pollution, are recognized as a significant, ongoing, global issue^1–4^. The deep sea comprises over 75% of the world’s inhabitable space for metazoans ^5^ and is key in sustaining biogeochemical cycles and ecosystem services^6,7^, vitally driven by the diverse benthic organisms living there^8–10^. In order to evaluate short- and long-term effects, data on the baseline biodiversity, its quantitative distribution, and the ecology of various species are essential. Though underwater monitoring methods using cabled or battery-powered observatories, optical cameras, sensors, and acoustic devices have been developed^11–13^, there are still many unanswered questions, especially below the seafloor. Most of the deep seafloor consists of flat, muddy plains argued as a major diversity sink, mostly consisting of infauna^6^. While distributions of large epifauna can be evaluated using such observatories or submersibles in the deep sea, infauna are hidden in the sediment and difficult to observe in situ.

Conventional sampling methods such as grab sampler or box corer do not have sufficient sampling area to quantify large infauna with low density, and dredge or trawl cannot quantify infauna accurately^14^. As an alternative, light- and radiation- based approaches (e.g., two-dimensional imaging of redistributed tracer particles^15^ and three-dimensional computed tomography (CT) imaging^16^) have also been used, where biogenic structures and traces of biological activities (e.g. burrows, pits, irrigation, feeding behavior) can be observed. Still, the tracer methods are two-dimensional with limited coverage area per measurement and although the CT method can reconstruct distributions in 3D it also has limited coverage area/volume and more importantly, cannot be applied for in situ monitoring studies.

Acoustic systems with various operating frequencies are commonly used for detecting objects buried in marine sediments, for example buried wooden shipwrecks under the seabed was visualized using chirp signals with 1.5–13 kHz swept pulses^17^. Recently, a new monitoring tool, named 3D acoustic “coring” system, was developed and used for the precise survey of buried plant roots with outer diameters of 5–10 cm using the ultrasound of 100 kHz center frequency^18^. Even higher frequency signals with center frequencies of 130–1000 kHz are starting to be used for laboratory-based tests to reveal bivalves and other small buried objects, including soft gummy worms^19–22^. Yet, there are very few attempts of in situ benthic surveys using acoustics, and none in the deep.

In this study, we report the first in situ application of a 3D acoustic “coring” system in the deep sea to image large infauna in the sediment. The imaging was conducted at the Off Hatsushima hydrocarbon seep site in Sagami Bay, Japan^23^. This seep is dominated by a very high density of vesicomyid clams, especially the sister species *Phreagena soyoae* and *P. okutanii*^24^, energetically reliant on chemoendosymbiotic bacteria. These clams live buried in the sediment, and extend their feet to extract hydrogen sulfide supplies from the deeper part of the sediment while their siphons are exposed to the surface seawater^25^. Though the adults are semi-infaunal, their juveniles have been difficult to observe and quantify as they are completely infaunal (Supplementary Fig. 1a), and their life position in the sediment is also difficult to image. This, in addition to their relatively high expected abundance, juvenile vesicomyid clams represent a suitable target for the first application of our deep-sea acoustic “coring” system.

## Materials and methods

### Grain size determination

To investigate the grain size distribution of the field site and to select a suitable frequency, we used sediment core samples collected from the Off Hatsushima hydrocarbon seep site, Sagami Bay, Japan. These cores were collected using H-type push core samplers^10^ near the planned in situ imaging area—a dense vesicomyid colony. The core sample was sliced into separate subsamples at 1 cm intervals for analysis of grain size distribution using a laser granulometer (LA-960, HORIBA Ltd., Japan) installed at the Geological Survey of Japan, National Institute of Advanced Industrial Science and Technology (AIST).

### Laboratory experiments

Before the field survey, laboratory experiments were conducted to confirm the performance of bivalve detection while they are buried in the sediment. The laboratory experiments were conducted in a small pool (120 cm in diameter, 30 cm in depth). Three shell samples of the venerid bivalve *Meretrix* sp. filled with water inside (length c. 42 mm, width c. 33 mm, thickness c. 18 mm) and four shell samples of *Corbicula* sp. filled with water inside (length c. 16 mm, width c. 14 mm, thickness c. 10 mm) were buried in the sediment completely. The sediment and clam shell samples were placed in a small metal palette (41 × 29 × 7 cm) with the A-core-2000 set over the tank. This experimental set-up is shown in Supplementary Fig. 2 and Supplementary Table 1.

Additional laboratory experiments were conducted to confirm the acoustic characteristics of the vesicomyid clams which inhabit the planned in situ observation area, with the set-up shown in Supplementary Fig. 3. Vesicomyid shells and sediment samples were collected from the Off Hatsushima seep using a scoop sampler by DSV *Shinkai 6500*. The laboratory experiments were conducted in a small pool (122 cm in width, 122 cm in depth, and 30 cm in height). Four settings were prepared for the laboratory experiments; Sample A: articulated valves filled with seawater inside and top of the shell exposed on the surface, Sample B: same as Sample A but completely buried, Sample C: single valve buried into the sediment, and Sample D: articulated valves filled with sediment and then completely buried into the sediment. The sediment and clam shell samples were placed in a small glass tank (15 × 15 × 15 cm) and the A-core-2000 was set over the tank. The thickness of the sediment was about 11 cm. All settings and parameters of the A-core-2000 were identical to the in situ observations.

### Field observation

**Figure 1:**
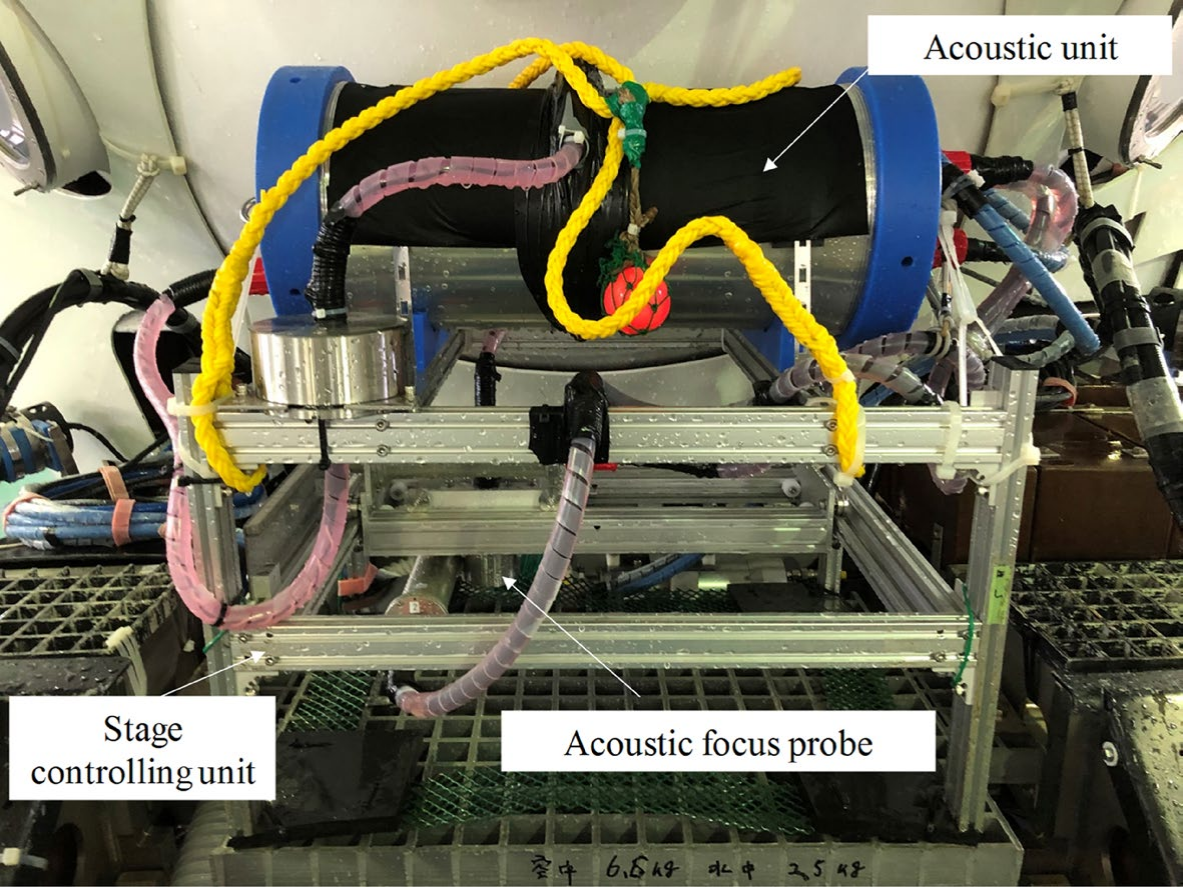
Deep-sea acoustic “coring” system (A-core 2000). The focused transducer is equipped on the pole extended form the 2D frame. It moves in horizontal directions and scans the sediment below.

We imaged the deep-sea sediment in situ in 2021 by deploying the acoustic “coring” system (named A-core-2000; Supplementary Fig. 1b) using the manned submersible DSV *Shinkai 6500*. Backscatters from the sediment and benthic organisms were observed using an automatic measurement system. The system mainly comprised a two-dimensional (2D) waterproof stage controlling unit (Arc Device, Koganei, Japan, depth rating 2000 m) and an acoustic unit (Japan Probe, Yokohama, Japan), as shown in Fig. 1. The stage frame was 640 mm in width, 500 mm in depth, and 637 mm in height. Acoustic transducers were attached to the front edge of an extended pole. The moving step of the stage varied from 1 to 250 mm and the movement precision was 0.1 mm in both X and Y directions. Backscatters were received by an acoustic focus probe 40 mm in diameter with a focal distance of 70 mm (Japan Probe, Japan, depth rating 3,000 m).

**Figure 2:**
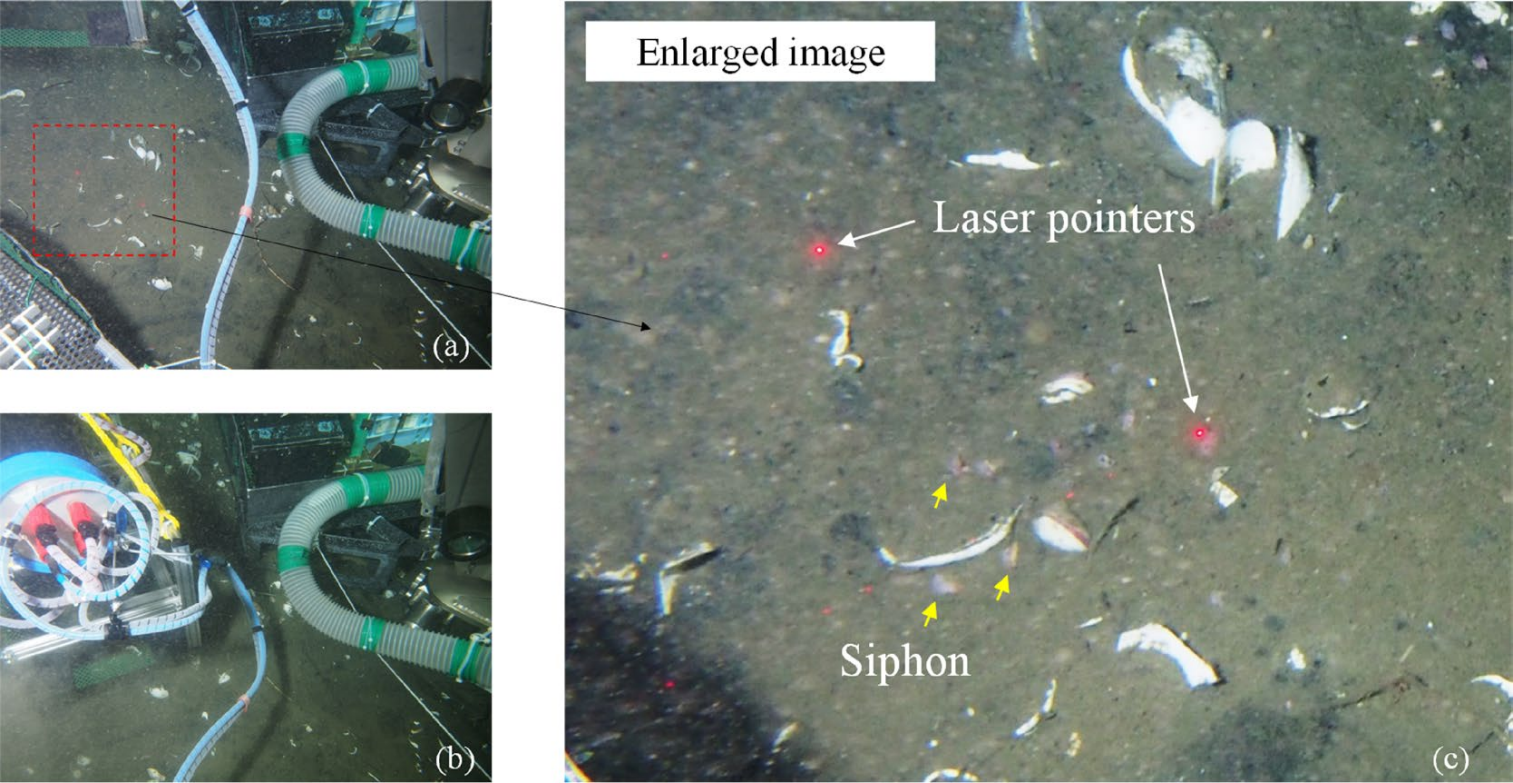
Deployment of the A-core-2000 on the seafloor. Laser pointers were used as markers and the system was gently placed (a → b). Several siphons could be seen on the seafloor surface (c).

Square pulse with a central frequency, selected according to actual habitat grain size measured as in “Grain size determination” section above (500 kHz), was generated by a pulser receiver in the acoustic unit and applied to the focus probe. The system automatically repeated the operations of stage moving, stage stopping, and acoustic measurements in the 2D area (X and Y). The area was set on the program before the measurement. Here, we set the measurement area to 250 mm in both X and Y directions, while the stage moved in this area at 2 mm step intervals. The scanning time was about 20 min. The in situ observation was carried out at the vesicomyid colony in the Off Hatsushima hydrocarbon seep site, Sagami Bay, Japan (depth 851–1237 m). We specifically targeted an area occupied by juvenile vesicomyid clams with only their siphons exposed on the surface, using laser pointers on the submersible as markers for the target position, and placed the system on the seafloor using a manipulator (Fig. 2). The distance between the probe and the sediment was set to around 100 mm at the origin of scanning. The backscatters were recorded on the acoustic unit with 10 MHz sampling rate. Each signal generated 3584 recorded data points. The 2D and 3D acoustic images were generated on the basis of waveforms after the recording. To visualize the space below the seafloor, a 3D acoustic image was reconstructed following the data processing flow which was presented in^18^ and briefly introduced in Supplementary Fig. 4. In this setting, the position accuracy of the target detection in the horizontal plane around the focus point was about 3 mm (Supplementary Fig. 5b,c).

## Results and discussion

For effective detection, it is important to determine a suitable frequency according to the size and depth of the detection target and the grain size of the sediment. The grain distribution of the core sampled from Off Hatsushima seep is shown in Table 1. The median grain diameter was around 0.052 mm and the 90% grain diameter was around 0.452 mm. The relationship between the grain size of the sediment and the frequency of ultrasound is well known, being observed and discussed in previous studies with laboratory experiments on absorption and scattering^16,26^. According to the theoretical model of the relationship^27,28^, ultrasound below 500 kHz is suitable for the survey in the case of silt and fine-grained sand with a grain size between 0.02 and 0.2 mm (Supplementary Fig. 6), matching the sediment grain size measured herein. In addition, a higher frequency generally results in higher resolution. Therefore, we decided to use the 500 kHz for the center frequency.

**Table 1:** Grain size distribution at the survey site.

Depth [cm]	Median diameter [mm]	10% diameter [mm]	90% diameter [mm]
0–1	0.055	0.009	0.455
1–2	0.089	0.008	0.558
2–3	0.087	0.008	0.566
3–4	0.065	0.008	0.420
4–5	0.062	0.007	0.571
5–6	0.019	0.007	0.262
6–7	0.018	0.007	0.389
7–8	0.020	0.007	0.397
Mean	0.052	0.008	0.452

**Figure 3:**
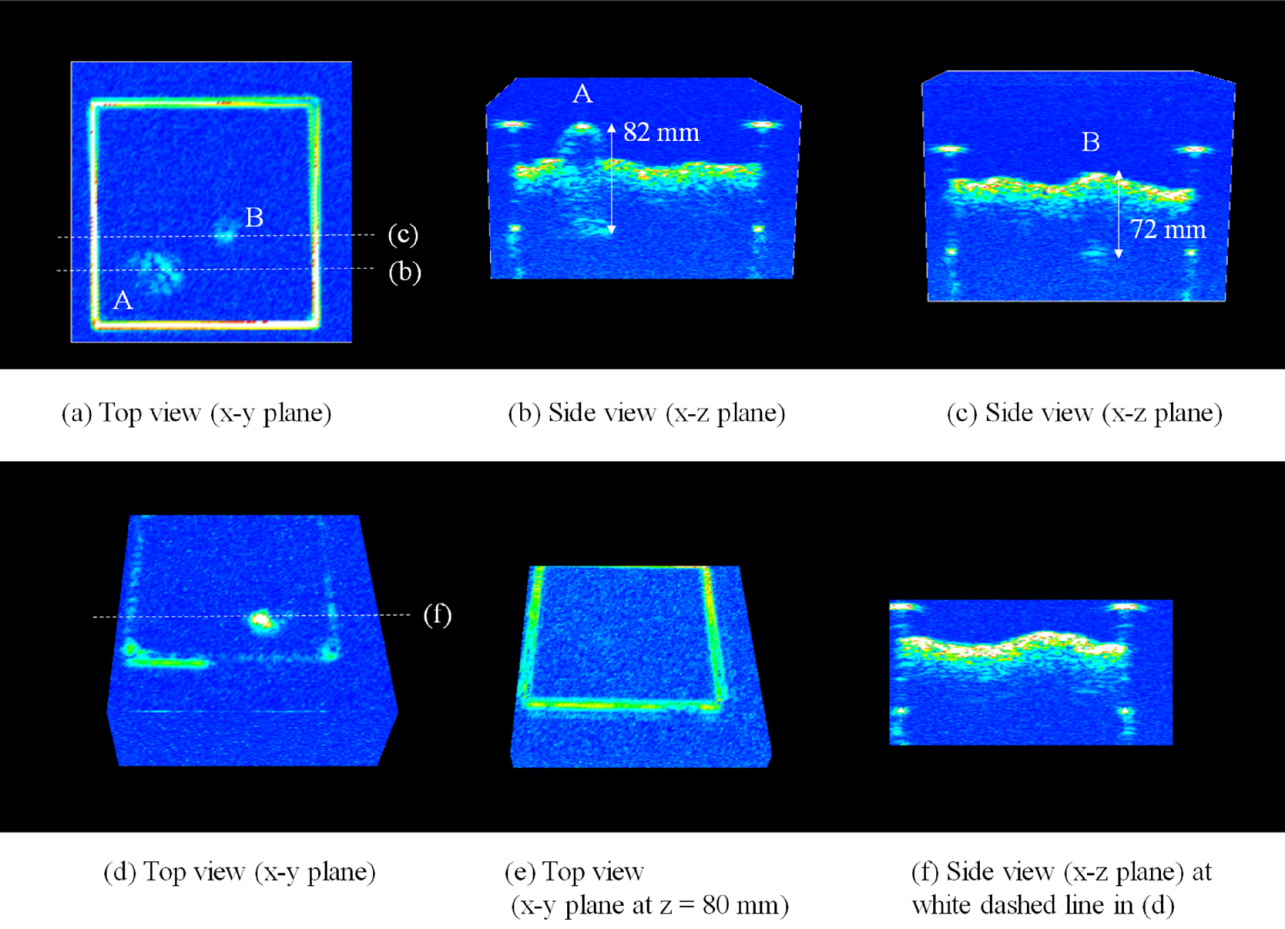
Cross-sectional acoustic images of the laboratory observations. (**a**–**c**) Experiment with Sample A and Sample B, whose reflectors are labelled A and B, respectively: (**a**) Top view showing the sediment surface. (**b**) Side view at the position of the white dashed line (**b**) in (**a**). (**c**) Side view at the white dashed line (**c**). (**d**–**f**) Experiment with Sample C and Sample D: (**d**) Top view around the sediment surface. (**e**) Top view at 80 mm depth from the sediment surface. (**f**) Side view at the white dashed line (**f**).

**Figure 4:**
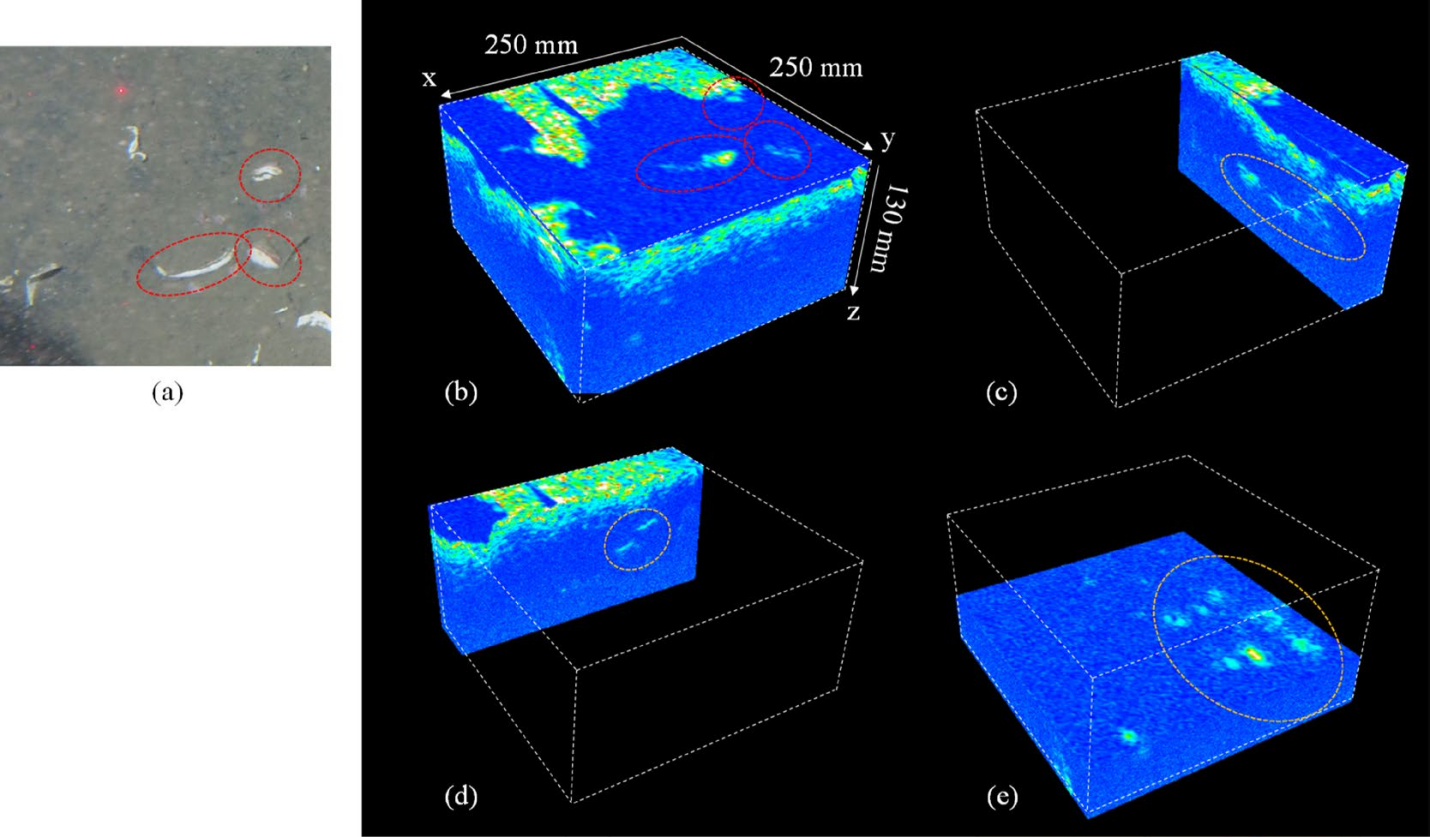
(**a**) Optical image of the seafloor at the observed area. (**b**) 3D acoustic image of the sediment. The position of the top surface is about 95 mm below the surface of the acoustic focus probe. (**c**) The cross-sectional view on the y–z plane at x = 40 mm. (**d**) The cross-sectional view on the x–z plane at y = 50 mm. (**e**) The cross-sectional view on the x–y plane at z = 79 mm. The same exposed empty valves can be seen in both optical (**a**) and acoustic (b) images (red ellipse dashed line). The strong backscatters can be detected at the cross-sectional views (yellow ellipse dashed line). The distance in z is estimated by the time difference with the sound speed of 1500 m/s as a reference.

**Figure 5:**
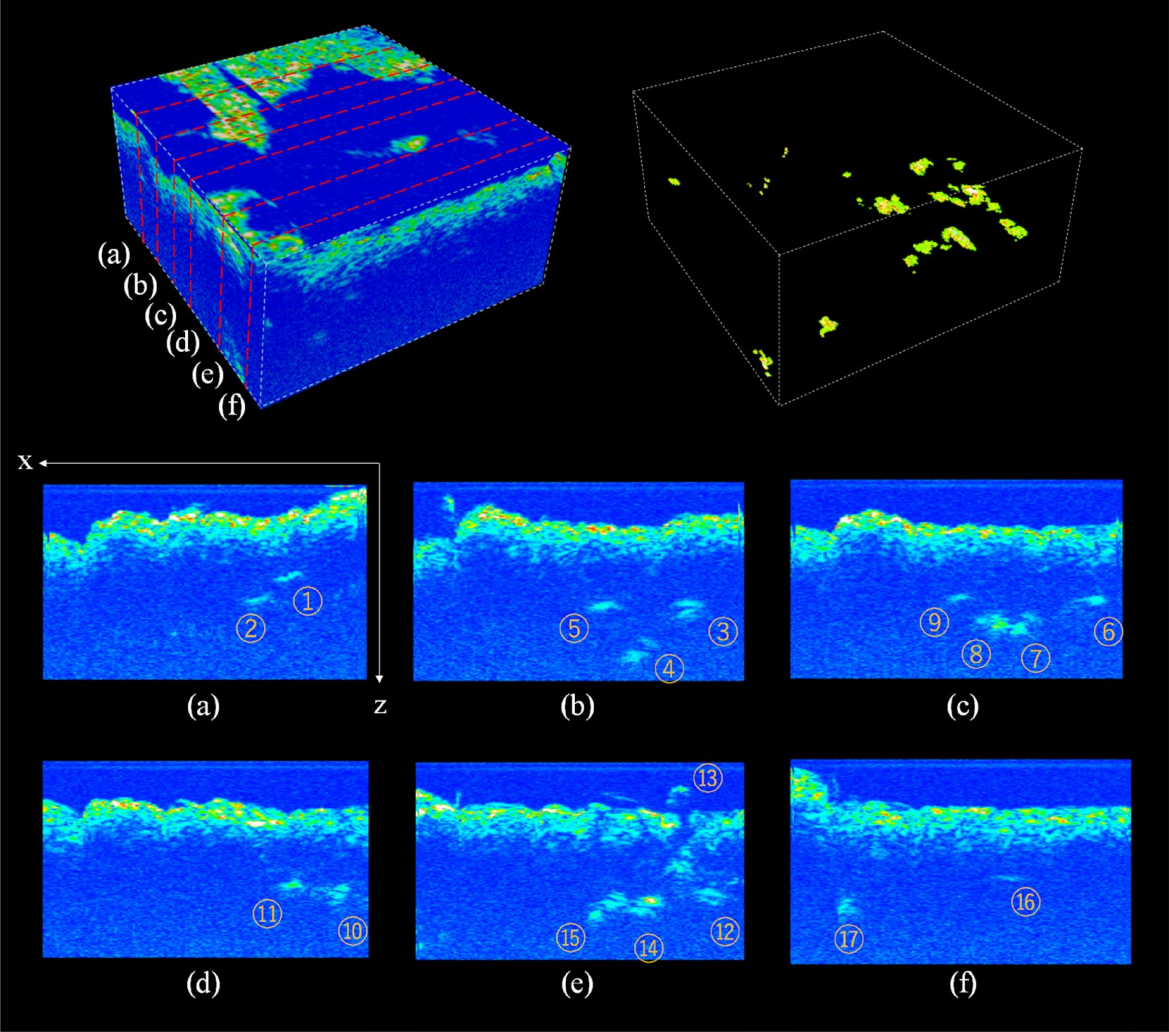
The cross-sectional views of the in situ imaging at x–z plane. (**a**) y = 50 mm, (**b**) y = 104 mm, (**c**) y = 134 mm, (**d**) y = 168 mm, (**e**) y = 204 mm, (**f**) y = 240 mm. The locations of the images are indicated in the top-left 3D acoustic image. Top-right image is a 3D rendering showing the locations of strong reflections below the surface with a certain threshold. The numbers (ID) are consistent with the ones in Supplemental Table 1.

The acoustic images obtained in the laboratory experiments are shown in Supplementary Fig. 7 and Fig. 3. All *Meretrix* and *Corbicula* shells were clearly detected in the acoustic images as shown in Supplementary Fig. 7, confirming that the method is able to detect signals from calcium carbonate shells. As for the in situ imaging data, strong backscatters were found below the sediment surface and 3D acoustic images were successfully reconstructed, visualizing the 3D space in the sediment with a voxel size of 1 mm^3^, as shown in Fig. 4. From the laboratory experiments with vesicomyid shells, the strong reflectors are shown to correspond to the lower end of the vesicomyid clam shells and were clearly observed in the articulated shells filled with water (Samples A and B; Fig. 3b,c), but no such reflection could be observed for sediment-filled shells or disarticulated valves (Samples C and D; Fig. 3e–f). This means reflection from the deeper end of the shell can be detected only when articulated shells are filled with water or similar medium. When alive, a large portion of the shell volume is occupied by seawater in the mantle cavity and the soft parts also have a low density closer to seawater than the sediment. As such, individual clams acoustically imaged with strong reflectors in situ are interpreted as being alive, or very fresh dead with articulated shells still filled by seawater. Figure 5 shows cross-sectional views of the in situ imaging where a total of 17 clear reflections from the lower end of shells were seen in the observed area (625 cm^2^), with approximate depths below the surface ranging between 41 and 98 mm (Supplementary Table 2). The positions of the reflectors appeared to correspond well to the positions where siphons were observed on the surface (Figs. 2 and 4a). The distance difference between the z position and the sediment surface position is likely useful as an approximation of the lengths of the individuals.

In the present study, we show that the A-core-2000 system can visualize large infauna with calcified exoskeletons inhabiting the deep-sea sediments non-destructively and non-invasively in their natural posture, exemplified by vesicomyid bivalves. With ultrasound of 500 kHz, we were able to selectively visualize live (or very fresh dead) vesicomyid clams in the Off Hatsushima seep, and 3D reconstruction of the acoustic data was able to shed light on their natural positions in the sediments. With a maximum resolution at the millimeter-scale, this approach is promising for the quantitative evaluation of deep-sea infauna with hard, calcified exoskeletons in the size range of larger macrofauna to megafauna, particularly in muddy bottoms such as abyssal plains. Moreover, the in situ living depth of the animals provides important insights into the structure and geochemistry of deep-sea sediments. Large infauna mix the sediment through locomotion or constructing burrows, thus playing important roles in determining sediment stability and structure^29^. Bioturbation by infauna also contributes to the relocation of fresh organic matter on the surface sediments, ultimately determining the fate of organic matter and biogeochemical cycles^10^. As prokaryotes, protists, and smaller animals rely on such mixed organic matter, surveying large infauna and their life positions using the A-core-2000 can provide crucial data in improving our understanding of how deep benthic ecosystems function.

Furthermore, the ability to carry out non-invasive observations makes acoustic “coring” a potential tool for bio-monitoring under environmental changes. Each scan with the A-core-2000 took approximately 20 min and can be done repeatedly, enabling time-series scanning per every 10 s of minutes to visualize the behavior of burrowing megafauna. The imaged area can be enlarged by repeating multiple measurements, and modifying the device for towed/mobile scanning is also a possibility in the future. According to the theoretical model in Supplemental Fig. 6, the 500 kHz signal was suitable for this survey area, but this can be changed to suit the specific habitat concerned. This can also be done for different depths of interest, as a lower frequency signal would be useful in visualizing the deeper parts of the sediment. Although the applicability to imaging soft-bodied infauna such as polychaete worms in situ remain unclear, recent laboratory experiments with soft materials such as gummy worms^22^ indicate that this may become possible in the future.

## Conclusions

Here, we propose a new approach based on acoustic techniques to visualize the 3D space in deep-sea sediments and successfully imaged burrowing bivalves in situ non-invasively, with potential applicability to other megafauna with calcified exoskeletons such as echinoderms and crustaceans. Three dimensional acoustic images were successfully reconstructed and the backscatters from infaunal clams were clearly found below the sediment surface. Our results support the effectiveness of the proposed acoustic system (A-core-2000) in detecting and imaging shelled infauna in situ and will be useful as a tool for understanding deep-sea infaunal biodiversity and biogeochemical cycles in a world rapidly impacted by anthropogenic activities, making sure the deep-sea infauna are no longer ‘out of sight, out of mind’.

## Supplementary Information

Supplementary Information.
