# Supplementary material for: Deep-sea infauna with calcified exoskeletons imaged in situ using a new 3D acoustic coring system (A-core-2000)
Source: Sci Rep. 2022 Jul 27;12:12101. doi: 10.1038/s41598-022-16356-3 (PMC9329462; doi:10.1038/s41598-022-16356-3)
Supplement: Supplementary file 1 — Supplementary Information. [file 41598_2022_16356_MOESM1_ESM.docx]

**Supplemental Information for:**

***Deep-sea infauna with calcified exoskeletons imaged in situ using a new 3D acoustic coring system (A-core-2000)***

**Katsunori Mizuno^1,2*^, Hidetaka Nomaki^2^, Chong Chen^2^, Koji Seike^3, 4^**

*^1^Department of Environment Systems, Graduate School of Frontier Sciences, The University of Tokyo,*

*Kashiwanoha, Kashiwa, Chiba 277-8561, Japan*

*^2^ X-STAR, Japan Agency for Marine-Earth Science and Technology (JAMSTEC), 2-15 Natsushima-cho, Yokosuka 237-0061, Japan*

*^3^ Geological Survey of Japan, National Institute of Advanced Industrial Science and Technology (AIST), Central 7, 1-1-1 Higashi, Tsukuba, Ibaraki, 305-8567, Japan*

*^4^ Department of Natural Environmental Studies, Graduate School of Frontier Sciences, The University of Tokyo, 5-1-5 Kashiwanoha, Kashiwa, Chiba, 277-8564, Japan*

***Corresponding author: Katsunori Mizuno**

**kmizuno@edu.k.u-tokyo.ac.jp**

(a)

(b)


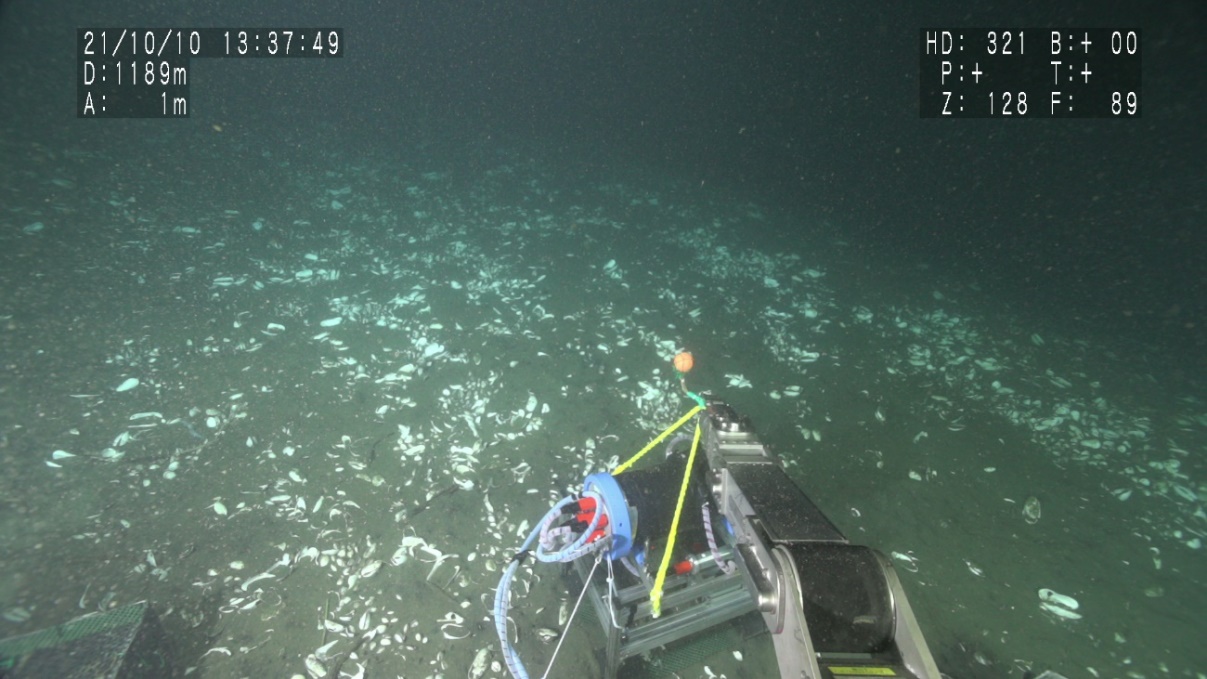


**Supplementary Fig. 1.** (a) Close-up view of vesicomyid clams. Juvenile clams live infaunally with only the siphon exposed on the surface. The photo was captured near the acoustic measurement area during the same dive. (b) A dense vesicomyid colony (mixture of dead shells and live colonies within approximately ~50 cm diameter) in the Off Hatsushima hydrocarbon seep site, Sagami Bay, Japan.

**Supplementary Fig. 2.** Photos of the laboratory experiments. Three *Meretrix* sp. (Me) and four *Corbicula* sp. (Co) were prepared for the laboratory experiments and then completely buried into the sediment.

**Supplementary Table 1. Grain size distribution of the sediment for the laboratory experiments.**

| Size [mm] | 0.425 | 0.3 | 0.212 | 0.15 | 0.106 | 0.075 | 0.053 | <0.053 |
| --- | --- | --- | --- | --- | --- | --- | --- | --- |
| % | 0 | 0.94 | 13.08 | 54.7 | 24.05 | 6.45 | 0.62 | 0.16 |

**Supplementary Fig. 3.** Photos of the laboratory experiments. The clam shell samples and sediment were collected with a scoop manipulated by DSV *Shinkai 6500* at Off Hatsushima, Sagami Bay, Japan.

**Supplementary Fig.4.** A 3D acoustic image was constructed and the space under the water bottom was visualized following this data processing flow. The spatial resolution of the image was 2 mm^3^/voxel. The origin is the start position of the measurement. Some broken lotus rods were found on the bottom surface. In this case, we adjusted the attenuation coefficient α for the image correction on GUI (Graphical User Interface) and set the value to 50 [dB/m].

**Supplementary Fig. 5.** The test for the position accuracy in horizontal plane onboard the *Shinkai 6500* (a). The plastic grid illustrated in (b) was measured by A-core-2000 in the deep sea (depth 1,000 m). The difference between actual grid and the acoustic image was around 3 mm in this test (c).

**Supplementary Fig. 6.** Attenuation coefficients with different grain sizes on the basis of the Biot – Stoll model and the multiple scattering model developed by Schwartz and Plona.

**Supplementary Fig. 7.** Cross-sectional acoustic images of the laboratory observations with *Meretrix* sp. (Me) whose reflectors are enclosed by the red squares Me1, Me2, and Me3 and four *Corbicula* sp. (Co) specimens whose reflectors are enclosed by red squares Co1, Co2, Co3, and Co4.

**Supplementary Table 2.** The coordinates of strong backscatters below the sediment surface interpreted as live (or very fresh dead) clams with articulated shells containing seawater or soft parts.

* The top of the shell was exposed above the sediment surface in this individual, and so this value indicates the position the top instead of sediment surface.
